# Who excels in online learning in Japan?

**DOI:** 10.3389/fpsyg.2025.1420996

**Published:** 2025-05-05

**Authors:** Eisuke Tokiwa

**Affiliations:** SUNBLAZE Inc., Tokyo, Japan

**Keywords:** Big Five personality traits, learning behavior in digital systems, online learning, self-regulated learning, COVID-19

## Abstract

**Introduction:**

This study aims to elucidate the impact of Big Five personality traits on learning behaviors and outcomes in online education. Given the increasing reliance on digital learning platforms, understanding individual differences in learning performance is crucial, particularly in the Japanese context.

**Methods:**

Data were collected from 103 third-year high school students in Tokyo, utilizing the online learning platform “Study Sapuri” and a personality traits questionnaire (BFI-2-J). Learning behaviors were assessed through system logs, while personality traits were measured using a validated psychological scale. Spearman correlation analysis was employed to examine the relationships between these variables. The correlation calculations were based on Equations 1-3, which provide the framework for assessing relationships between variables.

**Results:**

The analysis revealed that conscientiousness had the strongest positive influence on learning outcomes, with students scoring high in conscientiousness achieving superior results, particularly in STEM subjects. Additionally, agreeableness was positively associated with learning behaviors, such as the number of video content views on the platform. In contrast, extraversion showed varying effects depending on its facets: assertiveness positively influenced learning outcomes, while sociability was associated with a more passive approach. Moreover, students with high levels of neuroticism tended to adopt a cautious attitude toward learning, which was linked to longer study durations and potentially higher learning quality.

**Discussion:**

These findings highlight the role of personality traits in shaping online learning engagement and effectiveness. The study also suggests that Japan’s cultural context may influence the relationship between personality traits and academic performance, leading to outcomes for extraversion and neuroticism that differ from those observed in other countries. The implications of these results for personalized learning strategies and digital education design are discussed.

## Introduction

The Big Five personality traits are one of the most widely accepted personality models in psychology. This model explains human personality through five major traits: openness, conscientiousness, extraversion, agreeableness, and neuroticism (John and Srivastava, 1999). The Big Five personality traits are designed to comprehensively capture various aspects of personality, providing a powerful framework for understanding individual behavior, attitudes, and cognitive styles.

Openness refers to “the breadth, depth, originality, and complexity of an individual’s mental and experiential life” (John et al., 2008). Individuals with high openness are receptive to new experiences and ideas, imaginative, have a high interest in arts and culture, and tend to enjoy new challenges. They are often creative and possess unique perspectives. In contrast, individuals with low openness value tradition and are more resistant to new experiences. Openness is deeply involved in cognitive style and response to new experiences, with those high in this trait often exhibiting intellectual curiosity and enjoying complex problems (John et al., 2008). Additionally, they are more accepting of new ideas and cultural experiences, often having diverse perspectives and excelling in creative problem-solving (Schwaba et al., 2017). However, this trait may also show low adaptability to traditional values and rules (Gatzka and Hell, 2017). Openness is also associated with engagement in new activities in daily life and having unique hobbies, which allows individuals with high openness to welcome change and easily understand others’ different perspectives (Noftle and Robins, 2007).

Conscientiousness represents “socially prescribed impulse control that promotes task-and goal-directed behavior” (John et al., 2008). Individuals with high conscientiousness are self-disciplined, organized, responsible, strive to achieve goals, and act in a planned manner. They efficiently manage their time, pay attention to detail, and act according to plans. Highly conscientious individuals tend to set long-term goals and act in a planned manner to achieve them, excelling in self-management and time management skills (MacCann et al., 2009). This personality trait is crucial in professions and roles that require organized behavior and planning (Duckworth and Seligman, 2005). Additionally, conscientiousness strongly promotes behavior that adheres to social norms and rules, making highly conscientious individuals often reliable and trusted by others (Noftle and Robins, 2007). On the other hand, individuals with low conscientiousness tend to act impulsively, often lacking planning and self-management, resulting in inconsistent goal achievement and subsequently lower performance (Spengler et al., 2013).

Extraversion is characterized by “an energetic approach to the social and material world, including sociability, activity, assertiveness, and positive emotionality” (John et al., 2008). Individuals with high extraversion are sociable, enjoy interacting with many people, and act energetically. They gain energy from interacting with others, making them suitable for team activities and group work. Highly extraverted individuals often enjoy active social interactions and lead lively and energetic lives. They gain energy from interacting with others, tend to participate actively in group activities and events (Brandt et al., 2021), and extraversion facilitates smooth communication with others, enhancing leadership and teamwork skills (De Raad and Schouwenburg, 1996). On the other hand, individuals with low extraversion (introverts) prefer solitary activities and often engage in deep thinking and reflection. Introverted individuals find fulfillment in independent activities in quiet environments rather than deriving energy from social stimulation (Meyer et al., 2019).

Agreeableness is “a personality trait that reflects an altruistic, cooperative orientation toward others, in contrast to antagonism, and includes attributes such as altruism, warmth, trust, and modesty” (John et al., 2008). Individuals with high agreeableness are sensitive to others’ emotions and needs, value interpersonal relationships, and engage in cooperative behavior. Highly agreeable individuals are adept at building positive relationships with others and exhibit warmth and compassion in interpersonal interactions. As a result, they are often trusted by others and play a role in promoting cooperation and harmony within groups (Chamorro-Premuzic et al., 2007). Furthermore, agreeableness promotes empathetic and helpful behavior toward others, strengthening social connections. Those high in this trait tend to find fulfillment in helping others and are particularly successful in cooperative environments (Pawlowska et al., 2014). However, individuals with low agreeableness (antagonistic) may be competitive, assertive, and less sensitive to others’ opinions and emotions. Such traits can sometimes lead to conflicts and confrontations (Meyer et al., 2019).

Neuroticism is “a personality trait that contrasts emotional stability and calmness with negative emotionality such as anxiety, tension, sadness, and irritability” (John et al., 2008). Individuals with high neuroticism tend to be emotionally unstable and sensitive to stress, often perceiving their environment as more stressful (Ebstrup et al., 2011; McCrae, 1990). Highly neurotic individuals are sensitive to stressful situations and negative emotions and often find it difficult to cope with them. As a result, they are more likely to experience anxiety and tension in their daily lives (Hoferichter et al., 2014). On the other hand, individuals with low neuroticism (emotionally stable) are more likely to remain calm in stressful situations and, due to their high emotional stability, can effectively cope with difficult situations (Carver and Connor-Smith, 2010). Consequently, they typically have higher well-being and less anxiety and stress in daily life (Uliaszek et al., 2010). Additionally, individuals with high neuroticism are more likely to feel anxious in social situations and experience difficulties in interpersonal relationships due to their heightened sensitivity to others’ reactions and a tendency to anticipate negative outcomes (Barroso et al., 2021).

The Big Five personality traits have received widespread support in personality psychology research, with numerous studies investigating how each trait influences individual behavior and performance. For instance, openness plays a crucial role in learning and creative problem-solving, while conscientiousness is strongly associated with academic performance and workplace productivity (Costa and Mccrea, 1992). Understanding the Big Five personality traits is also valuable for improving performance in educational and workplace settings, enhancing mental health, and strengthening social relationships. For example, highly agreeable individuals tend to succeed in environments that emphasize teamwork, and highly extraverted individuals often excel in sales and customer service roles. Furthermore, individuals with high neuroticism can improve their quality of life by developing stress management and emotional regulation skills (McCrae and Costa, 1987). Thus, the Big Five personality traits provide a powerful tool for understanding various aspects of individual behavior and predicting behavior in different contexts. In this study, we explore how these Big Five personality traits affect the online learning performance of high school students. Specifically, we analyze the relationship between the Big Five personality traits and academic performance among high school students in Tokyo to examine the applicability of this model in East Asia.

## The Big Five personality traits and academic performance

The relationship between the Big Five personality traits and academic performance has been demonstrated in numerous studies. Conscientiousness, in particular, is widely recognized as the personality trait that most strongly influences academic performance (Poropat, 2009). Students with high conscientiousness tend to excel in exams and assignments due to their high self-management skills and their tendency to study in a planned manner (McCrae and Costa, 1992). According to Mammadov (2022) meta-analysis, conscientiousness shows a strong correlation with academic performance across all subject areas, with a particularly strong relationship with academic grades rather than standardized test scores. Some studies suggest that conscientiousness is especially important as a factor influencing teachers’ evaluations (Noftle and Robins, 2007; Trautwein et al., 2009), and its correlation with non-standardized assessments (e.g., classroom grades) may be higher (Brandt et al., 2021; Lechner et al., 2017). However, the effect of conscientiousness on standardized test scores has been reported to be inconsistent (Meyer et al., 2019; Spengler et al., 2013; Westphal et al., 2020).

Openness is also considered to be related to academic performance. For example, meta-analyses have shown a positive correlation between openness and academic performance (e.g., GPA and composite scores) (Mammadov, 2022; Poropat, 2009). Students with high openness are interested in new ideas and knowledge and tend to engage in deep reflection, which often leads to higher performance in creative assignments and exploratory learning (DeYoung et al., 2007). Furthermore, students with high openness may prefer intellectually stimulating situations, which may lead to higher performance on standardized tests (Schwaba et al., 2017; Willingham et al., 2002).

On the other hand, neuroticism is shown to have a negative impact on academic performance. Students with high neuroticism are sensitive to stress and anxiety, which can negatively affect their performance in exams and assignments (Hoferichter et al., 2014; O’Connor and Paunonen, 2007). Furthermore, high levels of test anxiety may lead to a negative impact of neuroticism on standardized tests (Byrne et al., 2015). However, students with high neuroticism may exhibit careful learning behaviors due to perfectionism (Smith et al., 2019), which may positively influence their grades. Teachers may also reflect neuroticism in their grading, suggesting that neuroticism might not always negatively impact academic performance.

Extraversion and agreeableness do not show consistent effects on academic performance but are suggested to influence social aspects of performance. Highly extraverted students tend to participate actively in group work and class discussions, which may enhance the quality of their learning experiences (Komarraju et al., 2011). The relationship between extraversion and academic performance may vary depending on the social characteristics of the learning environment (De Raad and Schouwenburg, 1996; Mammadov, 2022).

Students with high agreeableness tend to prioritize cooperation and support in interpersonal relationships and often perform well in collaborative tasks (Digman, 1990). They enjoy classroom discussions and cooperative activities, which may lead to higher evaluations from teachers (Chamorro-Premuzic et al., 2007; Pawlowska et al., 2014). However, this effect may be limited in standardized tests.

Subject-specific studies have shown that the impact of the Big Five personality traits on academic performance varies between language-related subjects and STEM (Science, Technology, Engineering, and Mathematics) subjects. For example, openness is particularly advantageous in language-related subjects, while its impact is limited in STEM subjects. Conscientiousness is strongly related to both subjects, and extraversion shows a stronger relationship in language-related subjects. Agreeableness is slightly advantageous in language-related subjects, and neuroticism tends to have a stronger negative impact, especially in STEM subjects (Meyer et al., 2024).

While most existing research has focused on university students, there are relatively few studies on the relationship between the Big Five personality traits and academic performance in high school students. According to Poropat (2009) meta-analysis, the Big Five personality traits consistently correlate with academic performance from elementary school through university, but detailed analysis at each educational stage is insufficient. Specifically, investigating the relationship between personality traits and academic performance in high school students may provide important insights for developing educational strategies and promoting personalized learning.

### The characteristics of online learning

Online learning has become an essential mode of education in the digital age. Online learning typically falls into two main formats: asynchronous learning and synchronous learning. In asynchronous learning, learners can progress at their own pace, accessing materials and lecture videos at any time. In contrast, synchronous learning requires participation in real-time online classes and group discussions (Hrastinski, 2008).

The advantage of asynchronous learning is its flexibility, allowing learners to study according to their schedules. This is particularly beneficial for students with busy schedules or adults balancing work and education. Additionally, learners can repeatedly access the material, allowing them to review until they achieve a deep understanding (Allen and Seaman, 2017). However, for learners with low self-management skills, there is a higher risk of delay or abandonment of studies.

Synchronous learning has the advantage of increasing motivation through real-time interaction. Learners can easily resolve questions and doubts by directly communicating with instructors and other students. Additionally, group work and discussions create a cooperative learning environment, providing a socially rich learning experience (Bernard et al., 2014). However, it offers less flexibility in terms of schedule.

Moreover, self-regulation is a crucial element in online learning. The attitude of proactively engaging in learning has been shown to significantly impact learning outcomes (Ryan and Deci, 2000). In an online environment, learners must independently manage their tasks and engage in self-regulated learning, requiring skills such as time management, self-assessment, and goal setting (Zimmerman, 2008).

The COVID-19 pandemic has further heightened the importance of online learning. As many schools suspended face-to-face classes and shifted to online education, the spread of online learning accelerated rapidly (Moorhouse, 2020). Numerous studies have been conducted on the effectiveness and challenges of online learning in this context. In this context, the Big Five personality traits have emerged as important factors in understanding individual differences in online learning adaptation and success.

A study conducted among 287 students in Germany who engaged in online learning at home during the COVID-19 pandemic showed a significant positive correlation between conscientiousness and academic performance and academic satisfaction (Rodrigues et al., 2022). A survey of 316 students in Turkey analyzed the relationship between personality traits and the use of online and blended learning, revealing a significant correlation between conscientiousness, self-efficacy, and the use of online learning (Alkış and Temizel, 2018). A study of 72 students in Israel examined the relationship between personality traits, self-regulated learning, and online learning, confirming a positive correlation between conscientiousness, emotional richness, creativity, loyalty, and online learning satisfaction (Cohen and Baruth, 2017). A survey of 92 students in Tunisia revealed that highly extraverted students are influenced by extrinsic motivation, highly conscientious students are consistently concerned about their performance, and highly open students are interested in others’ opinions and actively post on online discussion pages (Tlili et al., 2023). A study conducted with 553 undergraduate and 599 graduate students in China showed that students with high agreeableness, conscientiousness, and openness outperformed those with high extraversion and negative emotionality in online learning (Yu, 2021). When students self-selected the online learning environment, they were more likely to procrastinate, and the inhibition sub-factor of conscientiousness was found to influence non-procrastination behavior (Wang and Zong, 2023). A study on the relationship between motivation for online learning and the Big Five personality traits revealed that students with high conscientiousness are highly motivated when there is a high personal relevance, and those with high openness are strongly motivated by intrinsic factors such as pure interest and enjoyment in activities (Audet et al., 2021). A study analyzing students’ online discussions and final reports using Linguistic Inquiry and Word Count (LIWC) to assess Big Five personality traits found that conscientiousness and openness positively influenced online learning performance, with an analytical thinking style also being evident (Abe, 2020). A study comparing students divided into high and low negative emotionality groups showed that the high negative emotionality group had lower satisfaction with online learning (Baruth and Cohen, 2023).

These studies highlight the importance of the Big Five personality traits in online learning and serve as a critical foundation for understanding how personality traits influence learning outcomes. Based on these findings, this study analyzes the correlation between the Big Five personality traits and online learning performance in third-year high school students in Tokyo.

### The state of online learning in Japan

In recent years, with advancements in educational technology, the use of online learning applications has rapidly expanded. The COVID-19 pandemic, in particular, has brought significant changes to traditional education systems, accelerating the shift from in-person classes to online learning (Kanou, 2020). In response to the pandemic, the Ministry of Education, Culture, Sports, Science and Technology (MEXT) has promoted the “GIGA School Initiative,” which aimed to provide every student with a personal device. This initiative has facilitated the development of ICT environments at all educational levels, including high schools, further promoting the spread of online learning (Ministry of Education, Culture, Sports, Science and Technology (MEXT), 2022). As part of the GIGA School Initiative, the development of high-speed communication networks and the provision of communication devices for home study were advanced, rapidly promoting the digitization of education. As a result, the infrastructure for online learning was established, enabling students to continue high-quality learning even from home. This transition has led many schools to adapt to non-face-to-face learning, making online learning from home a common practice. The introduction of remote learning due to COVID-19, along with school closures, has not only significantly changed the learning environment but also brought new implications for the issue of school absenteeism.

According to (Ministry of Education, Culture, Sports, Science and Technology (MEXT), 2021), the number of students in national, public, and private elementary and junior high schools who were absent from school for extended periods reached approximately 299,000 in 2022, the highest on record. This increase is attributed not only to the impact of COVID-19 but also to various stress factors in school life. For instance, according to the “Survey on the Actual Situation of School Refusal (Including Trends)” conducted by the Shinshu Association of Free School Operators (2023), the factors contributing to school absenteeism include “problems related to relationships with teachers” (15.8%), “lethargy and anxiety” (12.8%), and “problems related to friendships, excluding bullying” (10.6%).

Furthermore, the “Survey on the Actual Situation of School Refusal Among Students and Families” conducted by the Shiga Prefecture Free School Network Association (2022) reported that the reasons for students feeling reluctant or wanting to take a break from school included “issues with teachers (not meeting teachers, being afraid of teachers, corporal punishment, distrust, etc.)” (23 cases), “issues with friends” (20 cases), “physical discomfort (such as headaches, stomachaches, and low-grade fever when trying to go to school)” (20 cases), and “feeling stressed by seeing teachers scold someone” (18 cases). These survey results indicate that interpersonal relationships within schools and physical discomfort are major factors contributing to school absenteeism.

As the number of students who are absent from school continues to increase, so does the prevalence of online learning from home. This trend can be attributed to the fact that more students who are unable to adapt to traditional school environments are opting for home-based learning. Online learning, which allows students to learn at their own pace, helps reduce stress associated with school life and makes it easier for them to engage in their studies. Additionally, by using recorded lesson content, students can repeatedly review material, deepening their understanding.

The advantages of online learning include flexible learning schedules, easy access, and the ability to customize learning content. For students who are absent from school, the ability to learn from home significantly reduces psychological burden and helps maintain their motivation to study. Among the many online learning applications, “Study Sapuri” is particularly popular in Japan, with a cumulative membership of 13,388,238 as of January 2023 (Infographics Study Sapuri 10th Anniversary, 2023). Furthermore, approximately 30% of high schools in Japan (1,614 out of about 5,000) have introduced this application. The introduction of this app has helped many students improve their basic academic skills. For instance, at Kunimi Elementary School in Fukui City, Fukui Prefecture, more than 70% of students reported that “the frequency of study increased” and that they “developed a habit of studying” (Komiyama, 2016).

Additionally, MEXT recognizes the importance of personalized learning (adaptive learning) tailored to the learning pace and non-cognitive abilities of individual students, and is promoting research and practice in this field (MEXT, 2021; Nishida et al., 2018). While Study Sapuri itself does not directly provide adaptive learning, its use serves as a tool to support individual learning.

### International and regional comparisons of personality traits

International and regional comparisons of personality traits are crucial for understanding the impact of cultural and social backgrounds on personality. The Big Five personality traits have been widely studied across various cultures, and numerous studies have reported differences in personality traits based on region (Schmitt et al., 2007). For example, differences in average scores of extraversion and agreeableness are often observed between Western countries and East Asian countries, leading to discussions on how these differences influence social behavior and academic performance. When focusing on the Japanese population, it is often reported that Japanese individuals tend to score lower in extraversion and agreeableness and have a particularly high level of neuroticism. Additionally, they are often found to score low in conscientiousness and openness.

In Western countries, individualism is typically emphasized, and autonomy and independence are highly valued. In contrast, East Asian countries emphasize collectivism, with a strong focus on social harmony and community consciousness (Triandis, 1995). These cultural backgrounds may manifest in different ways in the expression and influence of personality traits. For example, highly extraverted individuals may be encouraged to actively participate socially in Western contexts, whereas a more reserved attitude is often expected in East Asian contexts.

Moreover, the impact of personality traits on academic performance also varies depending on cultural background. For instance, Chen et al. (1995) compared students in the United States and China, reporting that Chinese students exhibited high conscientiousness and agreeableness, positively influencing their academic performance. In contrast, American students were found to have a strong correlation between openness and extraversion and their academic performance.

Mammadov (2022) meta-analysis also confirmed that geographical regions affect the relationship between personality traits and academic performance. Studies using Asian samples showed a higher average correlation coefficient for conscientiousness compared to other regions (*ρ* = 0.35), and strong correlations for agreeableness were observed in both Asian and Middle Eastern samples (*ρ* = 0.23 and *ρ* = 0.17, respectively). For extraversion, medium effect sizes were observed in Asian studies (*d* = 0.32), indicating a tendency for higher scores compared to other regions.

These international comparison studies highlight that the impact of personality traits on academic performance varies depending on culture and region. Based on this, the present study focuses on Japanese high school students and explores the relationship between personality traits and academic performance. Specifically, the study will examine the potential for regional differences in the outcomes of online learning.

This study hypothesizes that while conscientiousness will continue to positively influence academic performance, the high neuroticism characteristic of Japanese high school students may have distinct effects on online learning. Since high neuroticism does not necessarily correlate with lower academic performance, a careful analysis is required. To test this hypothesis, a correlation analysis of the Big Five personality traits and online learning performance will be conducted among third-year high school students in Tokyo. This will aim to clarify how personality traits affect online learning outcomes and contribute to the development of educational strategies that consider regional and cultural backgrounds.

### Challenges in existing research

Existing research faces several challenges. One major issue is sample bias. Many studies have focused on university students in Western countries, while research on students in East Asia, particularly high school students, is limited (Smith and Bond, 1999). This poses a challenge in accurately assessing the impact of cultural background and educational systems on the relationship between personality traits and academic performance. For example, while individualism is emphasized in Western cultures, collectivism is emphasized in East Asian cultures. Understanding how these cultural factors influence academic performance is essential (Markus and Kitayama, 1991).

Furthermore, while many studies target university students, research on high school students is relatively scarce. University students tend to have more advanced self-management and abstract thinking skills, making it difficult to compare their learning behaviors and performance with those of high school students. Research focusing on high school students is essential to understanding the impact of personality traits at an early stage of education and developing effective educational strategies (Eccles et al., 1993).

Lastly, when research findings are inconsistent, it is necessary to clarify the causes. For example, if results regarding the effects of extraversion or agreeableness differ, it is important to determine whether this is due to differences in samples, cultural factors, or measurement methods.

This study aims to address these challenges by conducting a correlation analysis of the Big Five personality traits and online learning performance among high school students in Tokyo. Through this, the study seeks to expand existing knowledge and contribute to the development of educational strategies.

### The Big Five personality traits and online learning among high school students in Tokyo, Japan

This study conducts a correlation analysis of the Big Five personality traits and online learning performance among third-year high school students in Tokyo. This choice is motivated by several reasons. First, as Japan’s capital, Tokyo attracts students from diverse backgrounds, making the results more generalizable. Moreover, Japan has a highly digitized educational environment, with many schools actively adopting online learning, making it easier to collect data on online learning.

The purpose of this study is to clarify the relationship between the Big Five personality traits and online learning performance among Japanese high school students. In particular, it aims to analyze in detail how conscientiousness, agreeableness, and neuroticism affect online learning outcomes. This will contribute to promoting personalized learning in Japan’s educational system.

The significance of this study lies in its ability to provide insights that will contribute to promoting personalized learning in educational settings by clarifying the relationship between personality traits and academic performance specific to Japanese high school students. In particular, curriculum design that takes into account differences in personality traits by country is necessary, and the introduction of curricula based on personality traits from other countries should be carefully considered. Priority should be given to constructing a learning environment based on Japan’s unique personality traits, and concrete methods for supporting learning that aligns with the personality traits of individual students should be developed. For example, for students with high conscientiousness, specific approaches to maximize the effectiveness of online learning should be considered. Meanwhile, for students with high extraversion, strategies to enhance social support should be prioritized, thereby improving the interactivity of online learning. In this way, this study aims to contribute to practical improvements in educational settings by analyzing in detail the relationship between personality traits and academic performance in Japan’s educational environment, thereby maximizing the effectiveness of online learning.

## Methods

### Sample and data collection

The determination of sample size was informed by previous studies investigating personality traits and academic performance, which showed sample sizes ranging from 72 to 316 participants (Cohen and Baruth, 2017: 72 participants; Rodrigues et al., 2022: 287 participants; Alkış and Temizel, 2018: 316 participants). Based on these benchmarks, we targeted a sample size of 100+ participants to ensure reliable results while considering practical constraints. The study was conducted at a high school in Tokyo, initially targeting all third-year students using Study Sapuri (*N* = 127). The final participation rate was 81.1%, resulting in 103 participants. This sample size was influenced by several practical factors, including the voluntary nature of participation, the requirement for both personality assessment and learning data availability, and time constraints of third-year students preparing for university entrance examinations. Data collection involved two main components. First, data on the usage of the online learning app “Study Sapuri” was collected from April 1, 2023, to December 31, 2023. Second, students completed the Japanese version of the Big Five Inventory-2 (BFI-2-J) (Yoshino et al., 2022) via Google Forms on December 20, 2023. The inventory consisted of 60 questions, each rated on a 5-point scale. Complete data on both Study Sapuri usage and personality traits were obtained from all 103 participants. Descriptive statistics for all variables in this study are presented in Table 1. All statistical analyses were performed using Python (version 3.6) in Visual Studio Code, with specific libraries including pandas and scipy.

**Table 1:** Descriptive statistics of all variables (*N* = 103).

Variable	Min	Max	*M*	SD
Openness to experience	1.83	5.00	3.45	0.61
Conscientiousness	1.00	4.50	2.77	0.69
Extraversion	1.33	4.92	3.21	0.83
Agreeableness	1.92	4.50	3.34	0.62
Neuroticism	1.17	5.00	3.06	0.89
Number of lectures watched	0	160.00	16.30	24.84
Viewing time(h)	0	71.14	7.85	9.51
Number of confirmation tests completed	1.00	623.00	86.75	89.97
Number of confirmation tests mastered	0	616.00	80.14	91.11
Average first attempt correct answer rate	0.21	1.00	0.62	0.12

### Study Sapuri

Study Sapuri provides recorded lecture videos, allowing students to learn at their own pace, anytime and anywhere, using a PC, tablet, or smartphone (Recruit Co., Ltd., 2023). The app offers flexible learning tailored to user needs, such as exam preparation, regular test preparation, or overcoming weaknesses. It is also possible to review and relearn basics from previous school years.

Study Sapuri offers over 40,000 lecture videos covering 19 subjects across six core areas necessary for elementary, junior high, and high school education, as well as university entrance exams. The videos are delivered by expert instructors and are divided into chapters of about 10 to 15 min each to help maintain concentration. The playback speed can be adjusted from the settings, and videos can be paused and resumed freely. Basic courses focus heavily on conceptual understanding, spending time on explanations to enhance students’ comprehension and improve understanding. At the end of each video, a “Confirmation Test” is provided, which is a web-based test to measure retention and help students overcome any difficulties. The confirmation test consists of multiple-choice questions, and the results are automatically graded and provided instantly.

Although students typically watch lecture videos at their own pace, teachers can also manage their learning. Teachers have access to a dedicated management interface where they can assign various homework tasks and easily check the results of both homework and independent study. The homework settings screen allows customization of the target student group, type of homework, distribution period, and comments. The progress management screen for each homework assignment enables teachers to monitor each student’s progress in detail. The results of the automatically graded confirmation tests can also be viewed in a list. The follow-up distribution function allows the teacher to extract students who achieve a certain correct answer rate and assign follow-up tasks to encourage review, providing tailored support for each student’s needs. The adaptive learning function identifies the “root cause of stumbling” and determines the “level of proficiency” based on past learning data, including homework. Study Sapuri then displays the most appropriate content for each student, facilitating personalized learning. In this study, the specific data items collected within Study Sapuri are as follows:

#### Number of lectures watched

The number of lecture videos viewed by the user. It is counted as one viewing when the video is fully watched.

#### Viewing time

The time spent by the user watching lecture videos, including partially viewed videos.

#### Number of confirmation tests completed

The number of “Confirmation Tests” completed at the end of each lecture video. These tests are designed to verify the user’s understanding of the content.

#### Number of confirmation tests mastered

This is counted as one when all “Confirmation Tests” at the end of all lecture videos within a unit group are completed.

#### Average first attempt correct answer rate

The rate of correct answers on the first attempt of each confirmation test, calculated as the number of correct answers divided by the total number of questions. The average of all first attempt correct answer rates is the Average First Attempt Correct Answer Rate.

### Spearman’s rank correlation coefficient

The data from 103 high school seniors were used to perform a correlation analysis between the Big Five personality traits and the use of StudySapuri (Number of Lectures Watched, Viewing Time, Number of Confirmation Tests Completed, Number of Confirmation Tests Mastered, Average First Attempt Correct Answer Rate). The Pearson correlation coefficient was used to evaluate the relationship between the Big Five personality traits and the use of StudySapuri. The Spearman correlation coefficient is defined as Equation 1, which quantifies the strength and direction of the monotonic relationship between two variables. Furthermore, Equation 2, 3 provide additional definitions for calculating the tied ranks and adjusted coefficients in cases where the dataset contains tied values.


(1) $$r=\frac{T_{x}+T_{y}-\sum_{i=1}^{n}{d_{i}}^{2}}{2\sqrt{T_{x}T_{y}}}$$

It is expressed as follows: However, the terms, and are determined from Equation 2, 3, represent the number of tied data in variables, respectively, while $t_{i}$ and $t_{j}$ for i, j = 1,2, …,103, denote the number of data included in each tied data. For tied data, the median of ranks is used.


(2) $$T_{x}=\frac{n\left(n^{2}-1\right)-\sum_{i=1}^{n_{x}}t_{i}\left({t_{i}}^{2}-1\right)}{12}$$

(3) $$T_{y}=\frac{n\left(n^{2}-1\right)-\sum_{i=1}^{n_{y}}t_{j}\left({t_{j}}^{2}-1\right)}{12}$$

### Correlations among Big Five personality traits

Openness showed a slight positive correlation with Conscientiousness, Extraversion, and Agreeableness, with correlation coefficients of 0.15, 0.15, and 0.17, respectively (Table 2). Conscientiousness exhibited a moderate positive correlation with Extraversion and Agreeableness (0.18 and 0.29, respectively), while it showed a slight negative correlation with Negative Emotionality (−0.18). Extraversion had a slight positive correlation with Agreeableness (0.13) and a moderate negative correlation with Negative Emotionality (−0.20). Agreeableness demonstrated a moderate negative correlation with Negative Emotionality (−0.21).

**Table 2:** Inter item correlation of BFI-2-J.

Big Five factor	1	2	3	4	5
1. Openness to Experience	—	0.15**	0.15**	0.17**	0.00
2. Conscientiousness		—	0.18**	0.29**	−0.18**
3. Extraversion			—	0.13**	−0.20**
4. Agreeableness				—	−0.21**
5. Neuroticism					—

**p < 0.01; *p < 0.05.

BFI-2-J refers to the Japanese version of Big Five Inventory-2, as described in the Methods section.

To examine the reliability of the Big Five personality trait scales used in this study, Cronbach’s alpha coefficients were calculated. The overall alpha coefficient for the scale was 0.75, indicating sufficient internal consistency. Furthermore, the alpha coefficients for each subscale were as follows: Openness (0.80), Conscientiousness (0.94), Extraversion (0.94), Agreeableness (0.91), and Neuroticism (0.96). These results indicate that each subscale also demonstrated high internal consistency, confirming the reliability of this scale. Based on these findings, it can be concluded that the Big Five personality trait scale used in this study possesses sufficient reliability both overall and within each subscale. Therefore, the data obtained using this scale can be considered to have the necessary quality for analysis.

### Effect sizes

The effect sizes for the Spearman correlations were classified using Cohen (1988) guidelines. Specifically, we categorized the effect sizes as follows: small (|*ρ*| = 0.10), medium (|*ρ*| = 0.30), and large (|*ρ*| = 0.50). This classification provides a standardized way to interpret the strength of the correlations observed in the study.

## Results

### Correlations between Big Five personality traits and study Sapuri “all subjects” usage data

To investigate how personality traits relate to overall online learning behavior and performance, we analyzed correlations between the Big Five factors and aggregated Study Sapuri usage metrics across all subject areas.

Conscientiousness showed a positive correlation with the number of Confirmation Tests Completed (*r* = 0.34, *p* < 0.001) and the number of Confirmation Tests Mastered (*r* = 0.35, *p* < 0.001) (Table 3). The subscales of Conscientiousness—Orderliness, Productiveness, and Responsibility—also demonstrated significant positive correlations with the number of Confirmation Tests Completed and Mastered (Orderliness: *r* = 0.20, *p* < 0.05; Productiveness: *r* = 0.33 ~ 0.34, *p* ≤ 0.001; Responsibility: *r* = 0.33 ~ 0.34, *p* ≤ 0.001). These findings suggest that students with high Conscientiousness tend to actively engage with Study Sapuri’s Confirmation Tests and achieve high performance.

**Table 3:** Results of spearman correlation coefficient in all subjects.

Big Five factor	Number of lectures watched	Viewing time (h)	Number of confirmation tests completed	Number of confirmation tests mastered	Average first attempt correct answer rate
Openness to experience	0.05	0.08	0	0.04	0.10
Conscientiousness	−0.04	−0.07	**0.34**	**0.35**	−0.06
Extraversion	−0.17	−0.12	0.11	0.09	−0.14
Agreeableness	**0.23**	0.16	0.06	0.07	−0.12
Neuroticism	0.17	0.15	0.01	0	0.07

Significant correlations (*p* < 0.05) are bolded.

Agreeableness showed a positive correlation with the number of Lectures Watched (*r* = 0.23, *p* < 0.05). The subscale of Agreeableness, Respect, also showed a significant positive correlation with the number of Lectures Watched (*r* = 0.24, *p* < 0.05). Similarly, the Trust subscale of Agreeableness was positively correlated with the number of Lectures Watched (*r* = 0.22, *p* < 0.05). These results suggest that students with higher Agreeableness are more likely to actively watch lectures on Study Sapuri.

The Sociability subscale of Extraversion showed a negative correlation with the number of Lectures Watched (*r* = −0.23, *p* < 0.05) and the Average First Attempt Correct Answer Rate (*r* = −0.25, *p* < 0.05). On the other hand, the Assertiveness subscale of Extraversion was positively correlated with the number of Confirmation Tests Completed (*r* = 0.26, *p* < 0.01) and the number of Confirmation Tests Mastered (*r* = 0.25, *p* < 0.05). These results suggest that while students with high Sociability may be less inclined to watch lectures or take Confirmation Tests on Study Sapuri, those with high Assertiveness are more likely to actively engage with the tests.

### Correlations between Big Five personality traits and study Sapuri usage data across four subjects

The correlation results between the Big Five personality traits and Study Sapuri usage data for four subjects are presented in Table 4. This subject-specific analysis aimed to identify potential differences in how personality traits affect learning behaviors and outcomes across different academic domains.

**Table 4:** Results of spearman correlation coefficient by subjects.

Big Five factor	Number of lectures watched	Viewing time (h)	Number of confirmation tests completed	Number of confirmation tests mastered	Average first attempt correct answer rate
Japanese					
Openness to experience	−0.02	0	−0.02	0.01	−0.06
Conscientiousness	−0.03	−0.04	**0.30**	**0.33**	0.09
Extraversion	−0.10	−0.10	0.13	0.08	−0.04
Agreeableness	0.04	0.06	0.01	0.04	0
Neuroticism	0.17	0.11	−0.01	−0.04	0.02
Mathematics					
Openness to experience	0.09	0.09	0.00	0.01	−0.02
Conscientiousness	0.08	0.07	**0.27**	**0.29**	−0.02
Extraversion	−0.02	0.01	0.05	0.05	−0.09
Agreeableness	**0.25**	0.16	0.11	0.11	−0.10
Neuroticism	0.16	0.21	0.05	0.04	0.11
English					
Openness to experience	0.02	0.02	−0.05	−0.02	0.17
Conscientiousness	−0.01	−0.07	**0.29**	**0.31**	0.19
Extraversion	−0.06	−0.13	0.05	0.05	0.01
Agreeableness	0.18	0.11	0.07	0.06	0.06
Neuroticism	0.22	0.18	−0.03	−0.03	0.00
Science					
Openness to experience	0.13	0.09	0.07	0.11	0.04
Conscientiousness	−0.11	−0.14	**0.30**	**0.33**	0.06
Extraversion	−0.08	−0.08	0.09	0.08	−0.11
Agreeableness	**0.22**	0.17	0.06	0.06	−0.03
Neuroticism	0.09	0.15	0.06	0.04	0.06

Significant correlations (*p* < 0.05) are bolded.

Language Subjects (Japanese): Conscientiousness showed a positive correlation with the number of Confirmation Tests Completed (*r* = 0.30, *p* < 0.01) and the number of Confirmation Tests Mastered (*r* = 0.33, *p* ≤ 0.001). The subscales of Conscientiousness—Orderliness (*r* = 0.20 ~ 0.22, *p* < 0.05), Productiveness (*r* = 0.31 ~ 0.33, *p* < 0.01), and Responsibility (*r* = 0.28 ~ 0.32, *p* < 0.01)—also showed significant positive correlations with the number of Confirmation Tests Completed and Mastered. Additionally, Responsibility was positively correlated with the Average First Attempt Correct Answer Rate (*r* = 0.21, *p* < 0.05). These findings suggest that diligent, orderly, and productive students actively engage with Japanese subject Confirmation Tests and achieve high performance. Extraversion (Assertiveness) showed a positive correlation with the number of Confirmation Tests Completed (*r* = 0.27, *p* < 0.01) and the number of Confirmation Tests Mastered (*r* = 0.23, *p* < 0.05), indicating that students with high Assertiveness are more proactive in taking Japanese tests.

Language Subjects (English): Conscientiousness showed a positive correlation with the number of Confirmation Tests Completed (*r* = 0.29, *p* < 0.01) and the number of Confirmation Tests Mastered (*r* = 0.31, *p* < 0.01). Orderliness (*r* = 0.20, *p* < 0.05), Productiveness (*r* = 0.25 ~ 0.26, *p* < 0.05), and Responsibility (*r* = 0.29 ~ 0.30, *p* < 0.01) also showed significant positive correlations with the number of Confirmation Tests Completed and Mastered, with Productiveness also positively correlating with the Average First Attempt Correct Answer Rate (*r* = 0.21, *p* < 0.05). This suggests that diligent, orderly, and productive students are also proactive in taking English subject Confirmation Tests. Negative Emotionality was positively correlated with the number of Lectures Watched (*r* = 0.22, *p* < 0.05), and Anxiety (a subscale of Negative Emotionality) was positively correlated with both the number of Lectures Watched (*r* = 0.29, *p* < 0.01) and the Viewing Time (*r* = 0.24, *p* < 0.05), suggesting that students with high Negative Emotionality tend to watch more English lectures for longer durations. Extraversion (Sociability) showed a negative correlation with Viewing Time (*r* = −0.20, *p* < 0.05), indicating that students with high Sociability tend to spend less time watching English lectures. Agreeableness (Trust) showed a positive correlation with the number of Lectures Watched (*r* = 0.22, *p* < 0.05), suggesting that students with a higher tendency to trust others are more proactive in watching English lectures.

STEM Subjects (Mathematics): Conscientiousness showed a positive correlation with the number of Confirmation Tests Completed (*r* = 0.27, *p* < 0.01) and the number of Confirmation Tests Mastered (*r* = 0.29, *p* < 0.01). Productiveness (*r* = 0.30 ~ 0.32, *p* < 0.01) and Responsibility (*r* = 0.28 ~ 0.30, *p* < 0.01) also showed significant positive correlations with the number of Confirmation Tests Completed and Mastered, suggesting that diligent and productive students actively engage in mathematics Confirmation Tests. Agreeableness showed a positive correlation with the number of Lectures Watched (*r* = 0.25, *p* < 0.05), indicating that students with high Agreeableness are more proactive in watching mathematics lectures. The subscale of Agreeableness, Respect (*r* = 0.27, *p* < 0.01), also showed a significant positive correlation with the number of Lectures Watched. Extraversion (Assertiveness) showed a positive correlation with the number of Confirmation Tests Completed (*r* = 0.21, *p* < 0.05) and the number of Confirmation Tests Mastered (*r* = 0.20, *p* < 0.05), suggesting that students with high Assertiveness are also proactive in taking mathematics tests. Negative Emotionality showed a positive correlation with Viewing Time (*r* = 0.20, *p* < 0.05), with Anxiety (a subscale of Negative Emotionality) showing a positive correlation with Viewing Time (*r* = 0.20, *p* < 0.05). This suggests that students with high Negative Emotionality tend to spend more time watching mathematics lectures.

STEM Subjects (Science): Conscientiousness showed a positive correlation with the number of Confirmation Tests Completed (*r* = 0.30, *p* < 0.01) and the number of Confirmation Tests Mastered (*r* = 0.33, *p* ≤ 0.001). Productiveness (*r* = 0.32 ~ 0.34, *p* ≤ 0.001) and Responsibility (*r* = 0.26 ~ 0.29, *p* < 0.01) also showed significant positive correlations with the number of Confirmation Tests Completed and Mastered, suggesting that diligent, productive, and responsible students are proactive in science tests. Agreeableness showed a positive correlation with the number of Lectures Watched (*r* = 0.22, *p* < 0.05), indicating that students with high Agreeableness are more proactive in watching science lectures. Respect (*r* = 0.22, *p* < 0.05) and Trust (*r* = 0.24, *p* < 0.05), subscales of Agreeableness, were also associated with the number of Lectures Watched, suggesting that students with high Agreeableness tend to have a keen interest in science lectures.

## Discussion

### Interpretation and significance of results

This study analyzed the correlations between the Big Five personality traits and usage data from the online learning app “Study Sapuri” among third-year high school students in Tokyo. The results suggest that the traits of Conscientiousness, Agreeableness, Extraversion, and Neuroticism each have varying impacts on online learning outcomes, consistent with previous research on personality traits in educational contexts (Poropat, 2009; O’Connor and Paunonen, 2007).

Firstly, Conscientiousness was confirmed as a particularly important personality trait in online learning, aligning with prior studies (Mammadov, 2022; Rodrigues et al., 2022). Conscientiousness showed a strong positive correlation with the number of Confirmation Tests Completed and the number of Confirmation Tests Mastered in Study Sapuri. This indicates that students with high Conscientiousness tend to actively engage in confirmation tests and achieve better results, supporting previous findings about the role of Conscientiousness in academic achievement (MacCann et al., 2009; Spengler et al., 2013).

Agreeableness also showed a positive correlation with the number of Lectures Watched in Study Sapuri. This finding aligns with research suggesting that students with higher Agreeableness are more likely to engage actively with learning materials (Chamorro-Premuzic et al., 2007; Pawlowska et al., 2014). Specifically, the subscales of Respect and Trust within Agreeableness suggest that students with higher Agreeableness are more likely to actively watch lectures.

For Extraversion, different subscales yielded different results, consistent with the complex relationship between Extraversion and academic performance noted in previous research (De Raad and Schouwenburg, 1996; Komarraju et al., 2011). The Sociability subscale of Extraversion was negatively correlated with the number of Lectures Watched and the Average First Attempt Correct Answer Rate, while the Assertiveness subscale showed a positive correlation with the number of Confirmation Tests Completed and the number of Confirmation Tests Mastered.

Finally, Neuroticism showed a positive correlation with Viewing Time, a finding that adds nuance to previous research on anxiety and academic performance (Hoferichter et al., 2014; Smith et al., 2019). Students with higher levels of Anxiety, a subscale of Neuroticism, tended to watch lectures for longer periods. This suggests that students with high Neuroticism are more cautious in their learning and may repeatedly watch lectures until they thoroughly understand the content, reflecting findings about the relationship between perfectionism and academic engagement (Uliaszek et al., 2010).

The significance of this study lies in understanding the influence of personality traits on online learning, contributing to the promotion of personalized learning as emphasized in recent educational research (Zimmerman, 2008; Yu, 2021). The findings confirm that traits such as Conscientiousness and Agreeableness are factors that contribute to success in online learning, highlighting the importance of learning support based on personality traits.

### Relation to previous studies

The results of this study support previous findings on the relationship between the Big Five personality traits and academic performance, while providing new perspectives within the unique context of online learning. Additionally, the analysis of subject-specific results clarified the impact of personality traits on learning outcomes in language subjects (Japanese and English) and STEM subjects (Mathematics and Science).

Furthermore, this study provides important insights into the impact of cultural background on the relationship between personality traits and academic performance. Previous research has shown that the distribution and impact of personality traits vary by region and culture (Mammadov, 2022; Schmitt et al., 2007). Japanese high school students are known to exhibit different tendencies in traits like Extraversion, Agreeableness, and Conscientiousness compared to students from other regions. The results of this study emphasize the importance of considering cultural background in such analyses.

Japanese high school students generally exhibit lower levels of Extraversion, which can be attributed to a collectivist cultural background and societal norms that value modesty and humility (Triandis, 1995). However, this study found that the Assertiveness subscale of Extraversion positively impacts academic performance in online learning. Specifically, students with high Assertiveness performed better in the number of Confirmation Tests Completed and Mastered, particularly in STEM subjects. This suggests that even in Japan, students with high Assertiveness may achieve better outcomes in online learning environments, prompting a reevaluation of how Extraversion affects academic performance across different regions.

Conversely, the Sociability subscale of Extraversion did not consistently show a positive impact on academic performance, aligning with previous research. In language subjects, Sociability was negatively correlated with the number of Lectures Watched and Viewing Time, suggesting that students with high Sociability may be less engaged in online learning. This may reflect the possibility that Sociability does not always positively influence academic performance in Japanese culture.

Neuroticism is known to be generally high among Japanese high school students (Chen et al., 1995). While high levels of this trait are typically associated with negative academic outcomes, this study revealed a different aspect. Specifically, students with high Neuroticism showed a positive correlation between Viewing Time and subjects like English and Mathematics, indicating that they tend to spend more time studying these subjects. This suggests that students with high Neuroticism may enhance their learning quality by proceeding cautiously in online learning environments.

In Japanese culture, persistence and effort, even under stressful or anxious conditions, are often valued as virtues. This cultural factor may be one reason why students with high Neuroticism channel their anxiety into learning energy, leading to increased study time. This could explain the differing results from studies conducted in other countries regarding the negative impact of Neuroticism.

Conscientiousness and Agreeableness were also found to have positive effects on academic performance, consistent with existing research (Poropat, 2009; McCrae and Costa, 1992). Conscientiousness showed a strong positive correlation with the number of Confirmation Tests Completed and Mastered in both STEM and language subjects, while Agreeableness was associated with the number of Lectures Watched. These results support the role of Conscientiousness and Agreeableness in enhancing academic performance in the Japanese educational context as well.

Overall, this study provides new perspectives by considering the influence of Japan’s cultural and geographical background on the relationship between personality traits and academic performance. It offers insights into how Extraversion and Neuroticism specifically impact online learning in Japan, contributing to the development of region-specific educational strategies. This further advances personalized learning by considering personality traits.

### Limitations of the study

This study provided important insights into the relationship between the Big Five personality traits and online learning performance among Japanese high school students, contributing to the existing geographical bias in research. However, several limitations exist that should be considered to improve future research.

Sample Bias: Data were collected from third-year high school students in Tokyo, Japan. This allowed the addition of a Japanese case to existing research and helped correct geographical bias. However, the sample was limited to specific grade levels, ages, and educational levels, resulting in potential bias. For example, including students from different grade levels or educational stages (such as middle school or university) might yield different relationships between personality traits and academic performance. Additionally, research involving students from diverse regions and cultural backgrounds is needed.

Research Design Limitations: This study was based on a cross-sectional research design and did not employ a longitudinal approach, making it impossible to capture changes in personality traits and academic performance over time. Future longitudinal studies are necessary to understand how the relationship between personality traits and academic performance evolves over the long term. Moreover, the sample size of 103 participants is relatively small, which may affect statistical power. Existing studies have used sample sizes ranging from several dozen to around 600, with 100 participants considered on the smaller side. However, simply increasing sample size is not recommended as it may lead to “Questionable Research Practices,” potentially compromising research reliability and reproducibility.

Additionally, there are limitations regarding the Study Sapuri data collection method. While Study Sapuri provides detailed learning analytics, we lack information about the specific circumstances under which students engaged with the platform. For instance, we cannot determine whether students were fully focused during video lectures or multitasking, whether they received help from others when taking confirmation tests, or how their physical learning environment might have influenced their online learning behavior. Furthermore, the accuracy of viewing time measurements may be affected by instances where students left videos playing without actively watching them. These limitations in data collection methodology could impact the interpretation of the relationship between personality traits and learning behaviors.

Measurement Method Limitations: This study used self-report questionnaires to measure personality traits, which may introduce self-report bias. If participants choose socially desirable answers or are unable to accurately assess their own personality, the results may be skewed. Additionally, the Study Sapuri usage data relied on a specific platform, which is available in Japan but may limit comparisons with studies in other countries. If different learning tools or measurement tools are used, the consistency and comparability of the results could be compromised. Future research should adopt diverse measurement methods.

Theoretical Model Limitations: This study analyzed the relationship between personality traits and academic performance but did not fully consider environmental factors, such as family environment, school environment, and teacher influence, which may significantly impact the relationship between personality traits and academic performance. Comprehensive analysis that includes these factors could lead to a deeper understanding. Furthermore, this study did not account for other important variables, such as gender and the presence of learning disabilities, which may mediate the relationship between personality traits and online learning outcomes. Future research should incorporate these variables in multivariate analyses to gain a more precise understanding of the relationship between personality traits and academic performance.

### Future research directions

This study clarified the relationship between the Big Five personality traits and online learning performance among Japanese high school students, providing new insights into the influence of personality traits on academic outcomes. However, considering the study’s limitations, further development and deepening of research are required in future studies.

Collecting More Diverse Samples: Future research should collect more diverse samples. Including students from different age groups and educational stages (such as middle school or university) will help clarify how the relationship between personality traits and academic performance changes with age and learning experience. Additionally, conducting research in other regions of Japan or in countries with different cultural backgrounds will further elucidate the impact of geographical and cultural factors on the relationship between personality traits and academic performance.

Implementing Longitudinal Studies: While this study adopted a cross-sectional design, longitudinal studies are necessary to gain a deeper understanding of the relationship between personality traits and academic performance. By collecting data over an extended period, it will be possible to track how personality traits and academic performance evolve and how they influence each other, enabling the identification of causal relationships and the evaluation of the effectiveness of educational interventions.

Improving Measurement Methods: To minimize the impact of self-report bias, future research should introduce multiple measurement methods. For example, combining teacher or parent evaluations with behavioral observations could lead to more objective assessments of personality traits. Moreover, collecting data from different educational platforms and environments will facilitate comparisons with studies conducted in other countries, leading to more universal insights into the relationship between personality traits and academic performance.

Considering Environmental Factors: To gain a more comprehensive understanding of the relationship between personality traits and academic performance, research that considers environmental factors is essential. Examining how factors such as family environment, school environment, and teacher influence affect personality traits and how these are reflected in academic performance could lead to the development of more sophisticated educational strategies. This would provide further insights for promoting personalized learning.

Incorporating Multivariate Analysis: Future research should conduct multivariate analyses that consider other variables, such as gender and the presence of learning disabilities, which may mediate the relationship between personality traits and academic performance. This will enable a more nuanced understanding of how personality traits impact academic outcomes in real-world learning environments. These insights will form the foundation for providing more personalized learning support.

### Practical implications

The results of this study have several important implications for educational practice, supported by empirical evidence. First, the confirmation that Conscientiousness strongly influences academic performance highlights the importance of enhancing support to develop this trait. Specifically, providing structured guidance on goal-setting and teaching self-management skills can effectively support students in planning and following through on their studies (Zimmerman, 2008; MacCann et al., 2009). For example, implementing digital learning portfolios and progress tracking systems can help students visualize their learning progress and develop metacognitive skills (Duckworth and Seligman, 2005).

For students with high Agreeableness, research suggests implementing collaborative learning strategies through structured online discussions and peer feedback systems (Pawlowska et al., 2014). These students tend to deepen their learning through cooperation and communication with others, as demonstrated in previous studies of online learning environments (Yu, 2021). For students with low Agreeableness, implementing scaffolded social interaction activities and providing clear communication guidelines has been shown to help develop social skills and build confidence in collaborative settings (Chamorro-Premuzic et al., 2007).

Regarding Extraversion, our finding that Assertiveness positively influences academic performance suggests implementing specific active learning strategies. These include structured online debates, virtual presentations, and problem-based learning activities where students can express and defend their ideas (Komarraju et al., 2011). However, given that students with high Sociability may engage less in individual learning, implementing a balanced approach that combines independent study with interactive activities is crucial (De Raad and Schouwenburg, 1996).

For students with high Neuroticism, evidence suggests creating a supportive learning environment that reduces anxiety while maintaining academic rigor (Hoferichter et al., 2014). Practical strategies include providing clear assessment criteria, implementing regular low-stakes practice tests, and offering structured revision materials (Smith et al., 2019). Additionally, incorporating stress management techniques and regular check-ins has been shown to help these students manage their anxiety while maintaining their careful approach to learning (Baruth and Cohen, 2023).

These findings contribute to the development of personality-aware learning environments. By implementing educational strategies that consider individual personality traits, educators can create more effective and inclusive learning experiences (Mammadov, 2022). This approach aligns with recent research on adaptive learning systems and personalized education (Cohen and Baruth, 2017), suggesting that tailoring instructional methods to students’ personality traits can significantly enhance learning outcomes in online environments.

## Conclusion

This study aimed to clarify how the Big Five personality traits influence online learning behavior and outcomes among third-year high school students in Tokyo. The results confirmed that Conscientiousness is strongly related to learning outcomes, and that Agreeableness, Extraversion, and Neuroticism each influence specific learning behaviors and outcomes. Notably, the study highlighted the potential impact of Japan’s cultural background on the relationship between personality traits and academic performance, with different results for Extraversion and Neuroticism compared to studies in other countries.

These insights provide a useful foundation for educators to develop instructional methods tailored to each student’s personality traits. Specifically, creating learning environments that enhance Conscientiousness and Agreeableness, and providing individualized support for Extraversion and Neuroticism, are expected to contribute to improved learning outcomes in online learning environments.

However, the study has several limitations, and future research should focus on collecting more diverse samples, conducting longitudinal studies, and analyzing environmental factors. Improving measurement methods and incorporating multivariate analysis will also enable a more precise understanding of the relationship between personality traits and academic performance.

Ultimately, the findings of this study are expected to contribute to the construction of educational strategies that consider personality traits, providing a foundation for maximizing students’ learning outcomes through more effective learning support. The promotion of personalized learning based on personality traits in educational settings will likely further enhance the effectiveness of online learning.

## Data Availability

The original contributions presented in the study are included in the article/Supplementary material, further inquiries can be directed to the corresponding author.
